# Dilation and Constriction of Subjective Time Based on Observed Walking Speed

**DOI:** 10.3389/fpsyg.2018.02565

**Published:** 2018-12-21

**Authors:** Hakan Karşılar, Yağmur Deniz Kısa, Fuat Balcı

**Affiliations:** ^1^Department of Psychology, Koç University, Istanbul, Turkey; ^2^Department of Psychology, Özyeğin University, Istanbul, Turkey; ^3^Koç University Center for Translational Medicine, Istanbul, Turkey

**Keywords:** biological motion, speed, psychophysics, temporal bisection, time perception

## Abstract

The physical properties of events are known to modulate perceived time. This study tested the effect of different quantitative (walking speed) and qualitative (walking-forward vs. walking-backward) features of observed motion on time perception in three complementary experiments. Participants were tested in the temporal discrimination (bisection) task, in which they were asked to categorize durations of walking animations as “short” or “long.” We predicted the faster observed walking to speed up temporal integration and thereby to shift the point of subjective equality leftward, and this effect to increase monotonically with increasing walking speed. To this end, we tested participants with two different ranges of walking speeds in *Experiment 1* and *2* and observed a parametric effect of walking speed on perceived time irrespective of the direction of walking (forward vs. rewound forward walking). *Experiment 3* contained a more plausible backward walking animation compared to the rewound walking animation used in *Experiments 1* and *2* (as validated based on independent subjective ratings). The effect of walking-speed and the lack of the effect of walking direction on perceived time were replicated in *Experiment 3*. Our results suggest a strong link between the speed but not the direction of perceived biological motion and subjective time.

## Introduction

Given that accurate timing is essential for the preparation and execution of most motor responses (see Buhusi and Meck, 2005), it can be implicitly assumed that perception of time is highly accurate across situations irrespective of what is being timed. However, it has been shown that changes in a stimulus’ properties such as its size, brightness, numerosity or loudness can also modulate time perception (e.g., Thomas and Cantor, 1976; Xuan et al., 2007; Eagleman and Pariyadath, 2009). In line with theories that assume a shared mechanism for the perception of various magnitudes by adhering to a common representational metric (e.g., time, numerosity, space; Walsh, 2003), perceived time also changes in the same direction with the changes in other stimulus properties. In other words, as one perceptual dimension is experimentally increased (e.g., loudness) so does the perceived duration of that stimulus (e.g., Berglund et al., 1969).

The relationship between motion and time perception has also been well documented (Brown, 1995; Kaneko and Murakami, 2009), where an increase in speed can lead to overestimations of durations, and vice versa (Matthews, 2011). Since motion can inherently be defined in terms of *change* per unit time (Poynter, 1989), it has been theorized that the larger amount of change experienced by the timing agent whilst observing faster motion or higher temporal and spatial frequencies (i.e., events happening more frequently across time and the level of detail in a stimulus per degree of visual angle, respectively) may in fact act as a proxy for the passage of time, and therefore lead to the observed overestimation of durations (Brown, 1995; Kanai et al., 2006; Kaneko and Murakami, 2009).

A prominent information-theoretic approach to modeling these variations in timing behavior generally assumes an internal clock (Treisman, 1963; Gibbon et al., 1984) with three hypothetical components: (1) a pacemaker-accumulator unit which generates and counts pulses, (2) a reference memory unit where the total number of pulses representing the timed interval are encoded, and (3) a decision component which compares the current number of pulses in the pacemaker-accumulator unit (i.e., working memory) to a random sample drawn from the reference memory unit in order to arrive at a temporal judgment (Gibbon et al., 1984). Thus, depending on the task used, stimuli with higher speeds could, for instance, speed up the pacemaker, thereby leading to longer perceived durations as a result of a higher number of pulses being registered per unit time in the accumulator (Zakay and Block, 1997; Wearden, 1999). On the other hand, a similar stimulus may lead to inadvertent attentional lapses which may lead to some of the pulses not getting registered in the accumulator (e.g., Penney, 2003; Karşılar and Balcı, 2016), thereby leading to shorter perceived durations (for a review see Allman et al., 2014). Examples of increases in the pacemaker rate (in addition to those mentioned above) have been shown in response to fast click-trains presented before timing a duration (Penton-Voak et al., 1996), higher body temperature (Wearden and Penton-Voak, 1995), emotional stimuli (Droit-Volet et al., 2004), auditory as opposed to visual timing stimuli (Wearden et al., 1998), physical activity/motion (Sayalı et al., 2018), as well as those manifested in terms of drug effects (see Coull et al., 2011 for a review). On the other hand, variations in perceived time due to attentional modulation have generally been shown in *dual-task* paradigms (e.g., Thomas and Weaver, 1975) where attentional resources are directed away from timing (Fortin and Rousseau, 1987; Macar et al., 1994), as well as in the *oddball* paradigm, where the duration of unexpected stimuli are perceived longer than those that were expected in a given trial (e.g., Tse et al., 2004; see Brown, 2008 for a review).

The neural energy model of timing that does not contain a pacemaker-accumulator architecture also readily accounts for the stimulus-property dependent findings outlined earlier. For instance, based on the series of findings that showed reduced neural activity (known as the *repetition suppression*; e.g., Wark et al., 2007) and shorter duration estimates of repeated stimulus (Pariyadath and Eagleman, 2007), Pariyadath and Eagleman (2007, 2008) proposed that the strength of neural responses reflecting metabolic costs of neural information-processing (referred to as *neural energy*) could be the determinant of the perceived duration. In support of this claim, the manipulation of various stimulus properties that are known to lead to longer temporal judgments (e.g., flicker rate, brightness, size) are also known to lead to higher neural signals in the corresponding brain areas (for review see Eagleman and Pariyadath, 2009).

It has been suggested that disparate neural/cognitive systems might be recruited with regard to the perception of animacy vs. inanimacy as well as the biological plausibility vs. implausibility of the observed stimulus (Caramazza and Shelton, 1998; Peuskens et al., 2005; Blake and Shiffrar, 2007; Shi et al., 2010; Zago et al., 2011). As such, research on the relationship between perception of time and perception of motion has been further distinguished in relation to these variables. For instance, the presentation durations of still images of running postures are judged to have lasted longer compared to images of standing postures (Yamamoto and Miura, 2012), while timing of still images that imply human movement are more precise than those with no implied motion (Moscatelli et al., 2011). Relatedly, the presentation durations of still images which show more intense actions that imply having taken longer (and more effort) to complete (Nather and Bueno, 2011), or words implying an action with faster average speed (e.g., “gallop”; Zhang et al., 2014) are generally judged to have lasted longer compared to their counterpart experimental conditions (but see Orgs et al., 2011, for an alternate account).

On the other hand, based on the now-well-documented finding that perception of biological vs. non-biological stimuli recruits different neural structures (Downing et al., 2001; Giese and Poggio, 2003), still other researchers have shown that the modulation of perceived time induced by observing a moving stimulus is directly mediated by the biological nature/plausibility of the observed action (Watanabe, 2008; Wang and Jiang, 2012; Lacquaniti et al., 2014). Similar results have been obtained with stimuli showing animate (i.e., not implied) vs. inanimate motion in real time (e.g., Carrozzo et al., 2010; Carrozzo and Lacquaniti, 2013).

In addition to the information-processing model based “internal clock speeding up due to higher arousal” account outlined above, discussion of results demonstrating a temporal bias in response to changes in biological stimulus properties has adhered to higher-order sensory-motor processes (Yamamoto and Miura, 2012), such as an effect of cognitive embodiment of perceived stimulus properties on perceived durations (Droit-Volet et al., 2013; Zhang et al., 2014). These effects are thought to result from the simultaneous cortical “simulation” of observed actions (Nather and Bueno, 2011; Chen et al., 2013), the potential underlying structures of which employ mirror neurons (Cattaneo and Rizzolatti, 2009). In other words, the ease with which a participant can cognitively simulate the action being observed might act as a mediator in perceiving its temporal properties, where those actions with which the timing agent is more familiar (i.e., regular walking as opposed to a non-biological action) can induce a stimulus-dependent bias in interval timing (see section “Discussion”). The mechanisms associated with “biological motion” also appear to be specific to certain brain areas. For instance, posterior superior temporal sulcus, fusiform face area, and occipital face area (Grossman and Blake, 2002) as well as premotor frontal areas (Saygin, 2007) have been associated with the processing of biological motion. Although the middle temporal area (MT) sends input to some of these areas (e.g., superior temporal sulcus), its activity is not specifically modulated by the biological nature of motion (Grossman et al., 2000 - see also Grossman and Blake, 2002 for other differentiations).

Overall, these studies support a directional relationship between perception of motion and the perception of time. The timing mechanism appears to be susceptible to the perceived speed of actual movement, as well as the implied speed embedded within still images (i.e., no actual physical change per unit time). We hypothesized that the length of perceived durations would increase parametrically with increased observed walking speed. Consequently, smaller differences in walking speed (*Experiment 1*) were expected to lead to a less pronounced effect of walking speed on perceived time, as opposed to larger effects due to larger differences among levels of the same variable (*Experiments 2* and *3*). Moreover, we expected larger effects when participants timed forward walking as opposed to backward walking motion, in addition to observing higher precision with which durations are timed in the forward walking condition due to it being a more familiar form of motion (i.e., processed more readily) in comparison to backward walking (Moscatelli et al., 2011; Loeffler et al., 2017). While previous research has conclusively demonstrated an effect of low-level motion on time perception (e.g., Kaneko and Murakami, 2009), no study so far has utilized straight-forward representations of human motion through the use of stick-figure actions to test for presumably more readily embodied effect of motion on perceived durations. Using an easily-discernible type of walking motion allows for testing the robustness and the sensitivity of the reported effect of basic motion on time perception when it is embedded in high-level (i.e., biological) motion. Additionally, perceiving biological motion might typically take place at supra-second intervals, since multiple elements are presumably patched together over longer-than-sub-second intervals to perceive various elements of the observed motion, such as its speed, agency and intentionality. Below we describe three experiments, all of which utilized the temporal bisection task, which entails categorizing experienced durations as short or long based on their subjective similarity with the short and long reference durations. As such, all three experiments tested time perception at the supra-second level with a large number of participants in order to contribute to the generalizability of earlier effects to larger samples and different procedures. Our results show that the walking speed has a parametric effect on perceived durations, irrespective of the direction of motion (i.e., forward vs. backward), supporting the first of our hypotheses, and not the second one. Importantly, direction of walking motion was chosen in this study as a variable primarily for purposes of operationalizing the qualitative familiarity and unfamiliarity to type of motion (i.e., forward vs. backward walking; see Viviani et al., 2011; Maffei et al., 2014) and not to make inferences about the possibly differential timing of biological motion *per se*. In essence, we use the term “biological motion” not as a methodological term (i.e., point light displays) as originally suggested by Johansson (1973) but rather as life motion, namely the “visual motion that expresses any sort of aspect characteristic for the motion of living beings” (Troje, 2013, pg. 4).

## Experiment 1

### Methods

#### Participants

Thirty-four participants (11 male, *M*_age_ = 21.8) were tested in *Experiment 1*. Participants received 1 course credit for their participation in *Experiment 1*. All experiments were approved by the Institutional Review Panel for Human Subjects of Koç University and were in accordance with the Declaration of Helsinki. All participants provided written consent for their participation. Two participants in *Experiment 1* were excluded from the analyses due to more than two excluded fits (see section below “Data Analysis”).

#### Stimuli and Apparatus

**FIGURE 1:**
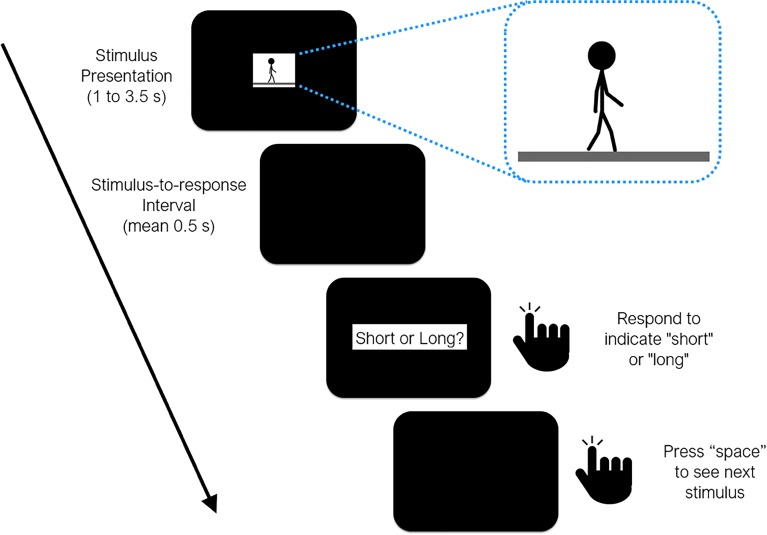
Timeline of events during a test trial in all three experiments reported in the study. Sample frame taken from the animation loop provided as inset (see Supplementary Materials).

Stimuli used in both experiments consisted of animations of a walking stick-figure (approx. height = 10 cm/9.5 visual deg. in diameter) composed of black lines for limbs and torso, as well as a black circle for the head (Figure 1; see Supplementary Materials for animations). The animations consisted of the stick-figure walking on a rectangular white background, which was placed on a black canvas that encompassed the entire screen. All stimuli and instructions were presented on a 21” LCD screen (60 Hz refresh-rate) on an Apple iMac G4 computer, generated in Matlab using the *PsychToolbox* extensions (Brainard, 1997). Participants sat at a distance of approximately 60 cm from the screen, in a dimly lit room with no chin-rest or other restrictions. Responses were provided by using a mechanical keyboard (Zalman ZM-K500).

One “cycle” of the walking animation consisted of two steps taken by the stick-figure, where the posture in the last frame was set so as to continue with the posture in the first frame, allowing us to perceptually wrap-around the walking motion to present it for as long as necessary. The center of the stick figure did not move on the x-axis, which gave the impression of a simultaneously moving camera at a right angle, while small movements of the body on the y-axis represented the characteristic bouncing motion as natural human walk. At 50 frames per second (*fps*), one cycle (i.e., two steps) lasted 1.5 s, which was considered to be the normal (baseline) speed of walking. Five distinct walking speeds were then produced by modulating the *fps* of the walking animation (40, 50, and 63 *fps*) each of which lasted for one of 6 probe durations (1.0, 1.5, 2.0, 2.5, 3.0, and 3.5 s). Hence, lower *fps* values led to slower and higher *fps* values led to faster walking speeds. Consequently, higher *fps* rates for a given duration, or longer durations for a given *fps* rate meant that more step cycles (albeit partially) were presented in the animation. Finally, mirror animations were prepared by rewinding (i.e., reversing) the walking action in each animation where the stick-figure walked backward, serving as the less plausible/less familiar walking condition.

#### Bisection Procedure

##### Training

Each session started with the presentation of two anchor durations at the offset of a space button press (short = 1 s, long = 3.5 s), represented by the presentation duration of a circular mottled texture (white, gray, black; 140, 10, 0.2 cd/m2 in brightness, respectively; approx. 8 cm/7.6 visual deg. in diameter). 10 training trials then ensued, in which the participants’ task was to report if the duration of the automatically presented circular texture was the short or the long one (5 random trials each). A trial was repeated if an incorrect categorization was given by the participant. The buttons denoting a “short” or a “long” response were randomly assigned in each session. Each participant attended a single session, which lasted 50–60 min. Participants were instructed not to count or use any other chronometric methods, which has been reported to be sufficient to prevent counting (Rattat and Droit-Volet, 2012).

##### Test

After 10 correct responses in the training trials, the experimental block commenced, in which the participants’ task was to categorize the six probe durations of walking animations as closer to the “short” or “long” anchor durations. Three walking speeds were employed: 40, 50, 63 *fps*. The animations started with the press of the space button. Once the animation ended, the participant was probed to respond after a stimulus-to-response interval sampled from an exponential distribution with a mean of 0.5 s and a lower bound of 0.2 s. All possible combinations of walking speeds (3 levels), probe durations (6 levels), and walking direction (2 levels) were randomly presented 12 times, leading to a total of 432 trials per session. No feedback was given after responding either for the reference or intermediate durations.

#### Data Analysis

**FIGURE 2:**
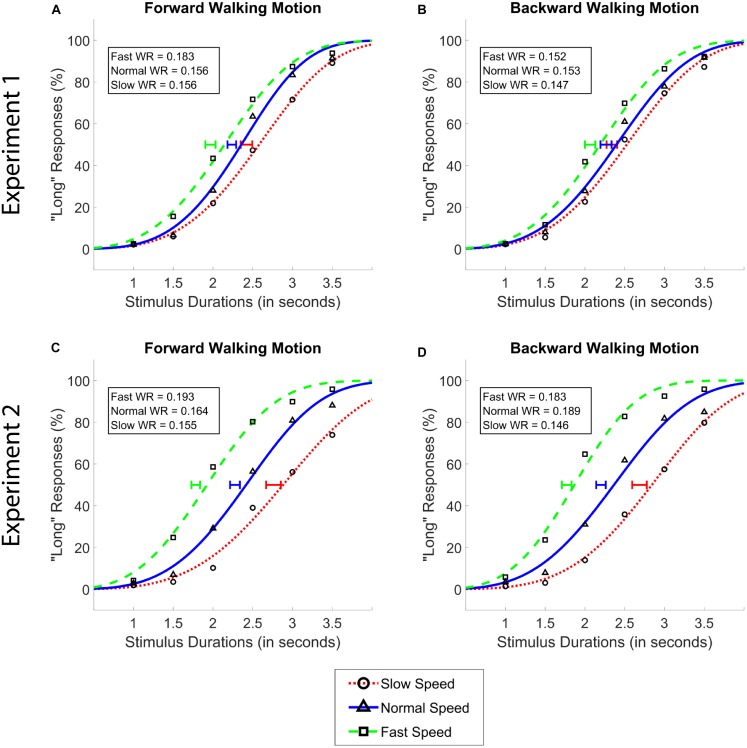
Average psychometric functions obtained by plotting the mean percentage of “long” responses as a function of probe duration in the forward **(A,C)** and the backward **(B,D)** walking conditions in Experiment 1 **(A,B)** and Experiment 2 **(C,D)**. Solid blue (triangle) lines denote “normal” walking speed, whereas dotted red (circle) and dashed green (square) lines denote slow and fast walking speeds, respectively. Standard Error of the Mean of individually calculated Points of Subjective Equality (PSE) are marked with horizontal lines of identical colors as the walking speed condition. Weber Ratios (WR) are provided as insets.

Mean percentage of “long” responses were plotted as a function of the six probe durations for each combination of walking speed and walking direction conditions, thereby forming six sigmoidal psychometric functions per participant (see Figure 2 – top panel for fits to average data). Standard two-parameter cumulative Weibull distribution functions were fit to these data. The parameters of fits with adjusted-R-squared values less than 0.70 (7% of the cases) were substituted by a random value that was drawn from the sample distribution. Points of subjective equality (PSE; the duration at which a short and a long response was equally likely) were calculated as the median of the Weibull fits. We were primarily interested in potential leftward or rightward shifts of the PSE values as a function of experimental conditions, which would typically be interpreted in terms of an increase or a decrease in clock speed (i.e., perceived time), respectively. We have also calculated the Weber Ratios (WR), which is a measure of the steepness of the psychometric function and refers to the sensitivity with which the probe durations are categorized. WR values were calculated by dividing the difference limen {[*p*(long) = 0.75 – *p*(long) = 0.25]/2} by the PSE. A higher WR value indicates that the participant had lower sensitivity (more difficult time) categorizing the durations as short or long.

A two-way repeated measures ANOVA with walking speed (3 levels; Exp 1: 40, 50, 63 *fps*; Exp 2: 25, 50, 100 *fps*) and walking direction (2 levels; forward and backward) as within subject factors, and PSE values as the dependent variable was conducted. In addition to conventional frequentist repeated-measures ANOVAs, we also report the results of these tests’ Bayesian counterparts for which we report inverse Bayes Factors (*BF*_10_; the strength of evidence the data provides for the alternative hypothesis compared to the null hypothesis; e.g., Wagenmakers et al., 2018b). *BF*_10_ values between 1–10, 10–100, and 100–300 are interpreted as providing weak to moderate, moderate to strong, and strong to decisive evidence for the alternative hypothesis, respectively. Conversely, the inverse of these factors (1/*BF*_10_) provide evidence for the null hypothesis in line with the same rule-of-thumb ranges (*BF*_01_; Jeffreys, 1961; Goodman, 1999); We used the JASP 0.9.0.1 open-source software with default priors for all Bayesian tests (see JASP Team, 2018; Wagenmakers et al., 2018a). In the manuscript, we indicate the model that has the highest Bayes factors with respect to the null model except for the model that contains an interaction, which is instead compared to the likelihood of the model that contains the main effects of the factors that were also included in the interaction model.

## RESULTS

Our analysis of data from *Experiment 1* showed a main effect of walking speed [*F*(2,62) = 47.04, *p* < 0.001, $\eta_{p}^{2}$ = 0.60], and no main effect of walking direction [*F*(1,31) = 0.34, *p* = 0.55], or an interaction between the two variables [*F*(1.57,48.52) = 2.34, *p* = 0.11, Greenhouse-Geisser Corrected]. *Post hoc* analyses showed that the difference between all walking speeds reached significance (*M*_Slow_ = 2.38, *M*_Normal_ = 2.25, *M*_Fast_ = 2.02; all *p*s < 0.01, see Figure 2). The Bayesian two-way ANOVA revealed an identical pattern of results where the data provided decisive evidence for the walking speed model over the null as the best model (*BF*_10_ > 300).

Identical repeated measures ANOVAs with WR as the dependent variable and walking speed and walking direction as the independent variables were conducted. In terms of their effects on WR values, neither the main effects nor their interaction reached significance (all *p*s > 0.05). The same pattern of results was observed with Bayesian analyses where the Bayes factors did not yield more than anecdotal evidence for any of the main or interaction effect models (all *BF*_10_ < 1).

## Experiment 2

We repeated *Experiment 1* with a new group of participants who were tested with a larger range of walking speeds. This allowed us to replicate the first experiment as well as to observe if larger differences between walking speeds indeed lead to larger differences in subjective time compared to *Experiment 1*. Additionally, by using unnaturally fast and slow movement speeds as timed stimuli, *Experiment 2* had the potential to reveal any effects of walking direction which may have been masked in the previous in the experiment, where fast and slow walking speeds were still within the interpretive limits of normal biological action.

### Method

#### Participants

Thirty-two participants were tested in *Experiment 2* (10 male, *M*_age_ = 21.2) and received 12 Turkish Liras for their participation.

#### Stimuli and Apparatus

The stimuli, apparatus, and the procedure used in *Experiment 2* were identical to those used in *Experiment 1* except for that participants were tested with 25, 50, and 100 *fps* in *Experiment 2* (as opposed to 40, 50, and 63 *fps* in *Experiment 1*). Data analyses were identical to *Experiment 1*. The parameters of fits with adjusted-R-squared values less than 0.70 (4% of the cases) were substituted by a random value that was drawn from a distribution with identical parameters as the sample distribution.

## RESULTS

Data from *Experiment 2* showed the identical pattern of results, with a larger size of the significant main effect compared to *Experiment 1*; namely a main effect of walking speed [*F*(1.34,41.64) = 105.44, *p* < 0.001, $\eta_{p}^{2}$ = 0.77], and no main effect of walking direction [*F*(1,31) = 2.32, *p* = 0.14], or an interaction between walking speed and walking direction [*F*(2,62) = 0.60, *p* = 0.55]. Again, identical with *Experiment 1*, *post hoc* analyses in *Experiment 2* showed that the difference between all walking speeds reached significance (*M*_Slow_ = 2.72, *M*_Normal_ = 2.24, *M*_Fast_ = 1.77; all *p*s < 0.001). Bayesian analyses affirmed these findings, where the data provided decisive evidence for walking speed as the best model over the null model (*BF*_10_ > 300).

Different from *Experiment 1*, a frequentist ANOVA revealed a significant main effect of walking speed on WR [*F*(2,62) = 7.48, *p* = 0.001] in *Experiment 2*. *Post hoc* analyses showed that WR values in the slow walking condition (*M* = 0.151) were significantly lower compared to the normal (*M* = 0.176) and fast (*M* = 0.188) walking conditions (both *p*s < 0.05), while the latter two conditions did not differ significantly from each other (*p* > 0.05). These findings were confirmed by the Bayesian ANOVA, which provided moderate evidence for the walking speed model over the null model (*BF*_01_ = 4.87) in terms of its effect on the WR values.

**FIGURE 3:**
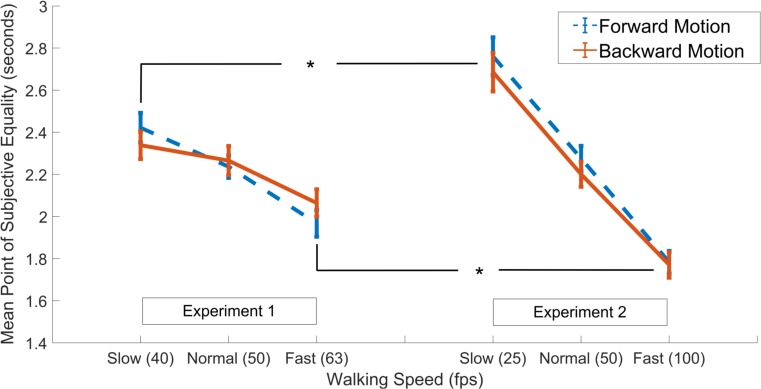
Results of independent samples *t*-tests comparing PSE values in the slow, normal and fast walking speed conditions in Experiment 1 (left panel) vs. Experiment 2 (right panel), separately for the forward (dashed blue lines) and backward (solid red lines) walking directions. Asterisks denote significant difference at the 0.05 level. Error bars show standard error of the mean.

Finally, we aimed to see if the degree of the effects were more prominent with larger differences in walking speed as manifested by the experimental paradigm (i.e., as in *Experiment 2* compared to *Experiment 1*). Thus, the data gathered in both experiments were subjected to a mixed ANOVA with walking speed and walking direction as two within-subjects factors, test group as the between-subjects factor (2 grouping levels; *Experiment 1* and *Experiment 2*), and PSE as the dependent variable. Results showed a main effect of walking speed [*F*(1.61,99.94) = 150.93, *p* < 0.001, $\eta_{p}^{2}$ = 0.71], in addition to an interaction between walking speed and the grouping factor [*F*(2,124) = 30.23, *p* < 0.001, $\eta_{p}^{2}$ = 0.33], while there were no main effects of walking direction or the grouping variable or any other interaction effects among the factors (all *p*s > 0.05; Figure 3). *Post hoc* independent samples *t*-tests showed that, in both the forward and backward walking conditions, the PSE values in the slow and fast walking speed conditions in *Experiment 2* were significantly lower and higher than those in *Experiment 1*, respectively (all *p*s < 0.05, Holm-Bonferroni corrected, see Figure 3), whereas there were no differences among the normal walking speed conditions in either direction (both *p*s > 0.05). Complementary analyses using the Bayesian method in a mixed-ANOVA setup revealed the strongest evidence for the interaction model between walking speed and the grouping variable among all models. This interaction model was decisively preferred to the walking speed and the grouping variable main effects model (both *BF*_10_ and *BF*_inclusion_ > 300).

## Experiment 3 and Stimulus Ratings

Previous research has shown that the detection of actual backward walking and digitally rewound forward walking require different types of cognitive competences (Viviani et al., 2011), and are likely perceived according to distinct perceptual factors and cognitive dynamics. This is evidenced by the fact that the simplest form of walking -in mechanistic terms-, is in fact a sequence of “falling” forward by shifting the center of the body mass, followed by a precisely timed catching of oneself before falling over or tripping, and then repeating this cycle with the contralateral limbs. As such, although walking backward utilizes the same mechanistic principle (i.e., temporarily shifting the center of mass, but this time backward), a different orchestration of a sequence of musculoskeletal coordination (and therefore a different observable movement) is utilized for relocating the body toward a backward position. Therefore, we conducted a third experiment to replicate *Experiment 2* with stimuli embedded with more plausible backward walking action animations compared to those used in *Experiment 1* and *Experiment 2*, in which backward walking animation was prepared by mimicking actual animations of forward moving profiles - see Supplementary Materials. We had these new animations rated by an independent group of participants in terms of how plausible they appeared.

### Method

#### Participants

Twenty-nine participants (11 male, *M*_age_ = 21.1) attended the *Stimulus Rating* experiment, and a different group of 25 participants were tested in *Experiment 3* (9 male, *M*_age_ = 21.8). Participants received 1 course credit in *Experiment 3* and 0.5 course credit for their participation in the *Stimulus Rating* experiments. All experiments were approved by the Institutional Review Panel for Human Subjects of Koç University. All participants provided written consent for their participation.

#### Stimuli and Apparatus

##### Stimulus Rating

Realistic backward walking animations were prepared using the same software methods used in *Experiments 1* and *2* for preparing forward walking animations, this time by observing animations of humans purposefully walking backward in an upright position. Separate backward walking animations were prepared in keeping with all walking speed (3 levels) and probe duration (6 levels) conditions used in *Experiment 2.* Stimulus presentation and response collection methods, as well as other experimental conditions were identical to those in *Experiments 1* and *2*.

##### Experiment 3

All apparatus and response collection methods were identical to those used in *Experiment 1 and 2*. All “rewound backward walking” stimuli from *Experiment 2* were replaced by the “realistic backward walking” animations in *Experiment 3* (see section above “Stimulus Rating”).

#### Procedure

##### Stimulus Rating

Each trial consisted of the presentation of two consecutive backward walking animations, each of which started at the onset of a space button press by the participant. On each trial, one of the animations was a backward walking animation generated by rewinding forward walking (as in *Experiment 1* and *2*), and the other was the novel, more realistic backward walking animation. The order of the animations was counterbalanced across trials. The two animations matched in their *fps* parameters (25, 50, or 100) and presentation durations (1.0, 1.5, 2.0, 2.5, 3.0, or 3.5 s). Combined levels of both variables (*fps* and duration) were counterbalanced and animations depicting each combination were presented twice in a single 20–25-min-long session. Presentation of the first animation was followed by a blank screen and an inter-stimulus interval (ISI) drawn from an exponential distribution with a mean of 0.5 s and a lower bound of 0.2 s. The ISI was followed by the written instruction to “Press the space button to see the second animation.” Immediately after the end of the second animation, the participant was asked to state which of the two animations (i.e., former or the latter) had the more plausible form of backward walking. The button press was followed by a response-to-stimulus interval with identical randomly distributed delays as in the ISI.

##### Experiment 3

All procedures, variables and parameters were identical to *Experiment 2*.

## RESULTS

### Stimulus Rating

Choice (as more familiar/plausible) proportions for the rewound backward and realistic backward walking animations were calculated. A one-sample *t*-test comparing choice proportions for the novel stimuli (over the rewound version) to chance level (i.e., 50%) revealed that the participants were significantly more likely to choose the novel backward walking stimuli over the rewound backward walking stimuli used in *Experiment 2* as more plausible [*M* = 58.3, *SD* = 19.1, *t*(28) = 2.34, *p* < 0.05] A Bayesian one sample *t*-test showed that the choice proportions for the novel stimuli were 2.03 times more likely than the chance level.

### Temporal Bisection Experiment

Identical analyses as those in *Experiment 2* were performed with identical inclusion/exclusion criteria and mean inoculation methods. The PSE and WR values were calculated for each participant by fitting standard two-parameter cumulative Weibull distribution functions to individual data. Identical with *Experiments 1* and 2, this procedure was carried out for each combination of walking speed and walking direction conditions.

**FIGURE 4:**
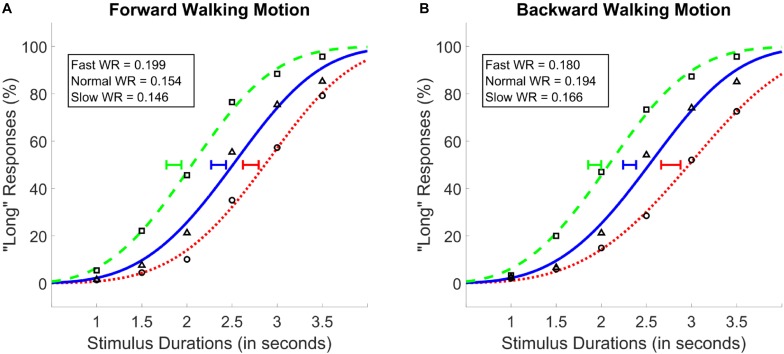
Average psychometric functions obtained by plotting the mean percentage of “long” responses as a function of probe duration in the forward **(A)** and the backward **(B)** walking conditions in Experiment 3. Solid blue (triangle) lines denote “normal” walking speed, whereas dotted red (circle) and dashed green (square) lines denote slow and fast walking speeds, respectively. Standard Error of the Mean of individually calculated Points of Subjective Equality (PSE) are marked with horizontal lines of identical colors as the walking speed condition. Weber Ratios (WR) are provided as insets.

A two-way repeated measures ANOVA with walking speed (3 levels; 25, 50, 100 *fps*) and walking direction (2 levels; forward and backward) as within subject factors, and PSE values as the dependent variable was conducted. Results showed a main effect of walking speed [*F*(1.34,32.2) = 89.38, *p* < 0.001, $\eta_{p}^{2}$ = 0.79, Greenhouse-Geisser corrected], and no main effect of walking direction [*F*(1,24) = 0.99, *p* = 0.34], or an interaction between the two variables [*F*(2,48) = 1.45, *p* = 0.26; see Figure 4]. *Post hoc* analyses showed that, identical with the results of *Experiments 1* and *2*, the difference between all walking speeds reached significance, where the fastest walking speed led to the lowest PSE followed by the normal and the slow walking speed conditions (*M*_Slow_ = 2.74, *M*_Normal_ = 2.33, *M*_Fast_ = 1.89; see Figure 4); There was a monotonic relationship between walking speed and PSE. As with Experiments 1 and 2, Bayesian ANOVAs with identical variables confirmed the results of the traditional ANOVAs such that the walking speed model was preferred against the null as the best model (*BF*_10_ > 300).

Identical repeated-measures ANOVAs with WR as the dependent variable and walking speed and walking direction as the independent variables were conducted. None of the main effects or the interaction between the variables was found to be significant (*p* > 0.05). These findings were confirmed by the Bayesian ANOVA, which provided no evidence for any of the main or interaction effect models (*BF*_10_ < 1).

## General Discussion

We conducted three experiments in which participants’ task was to categorize six durations of animations depicting a stick-figure, walking forward or backward, at three different walking speeds. The forward-backward walking direction was added to the current study as a variable in a similar vein to previous studies which assigned backward walking the unique property of representing “unfamiliarity” of a given biological motion (i.e., Viviani et al., 2011; Maffei et al., 2014), any natural form of which could be considered “familiar” unless artificially manipulated. The first two experiments differed only in the degree of difference between the faster and the slower walking speed. When data from the first two experiments were examined separately, as well as in conjunction, our results suggested that subjective time dilates with faster observed walking speed and it constricts with slower observed walking speed. On the other hand, the direction in which the stick-figure walked (forward or backward) did not have an effect on perceived time, and it did not interact with walking speed in any of the experiments. In a third experiment, we tested participants with a more naturalistic backward walking animation (as opposed to rewinding forward motion as in the first two experiments) and replicated these findings.

There are two primary mechanisms through which subjective time can be modulated within the “pacemaker-accumulator” theoretic framework; these are 1) changes in the pacemaker rate and 2) changes in the probability by which pacemaker signals are integrated in the accumulator (Penney, 2003). In relation to our experimental manipulation, faster walking speed can be assumed to either increase the pacemaker rate (e.g., due to arousal) or lead to a decrease in attention to time (e.g., due to divided attention) and vice versa for slower walking speeds. Under the first possibility (i.e., change in pacemaker rate), subjective time would be expected to dilate with faster walking speed, while the opposite predictions would be made if the effects were on attention to time. To this end, our results directly support the effect of observed walking speed on pacemaker rate. Importantly, walking speed had a parametric effect on clock speed; Compare the effect sizes in *Experiment 1* with *Experiments 2* and *3* with differential degrees of deviation between walking speeds (see Figures 3, 4).

Interval timing models which employ such a switch component also assert the possibility of stimulus effects on switch closure (timing onset) and opening (timing offset) latencies (Gibbon et al., 1984; Zakay and Block, 1995; Wearden et al., 1998). An increase in switch closing or opening latency would lead to under or over-estimation of perceived durations, respectively, whereas simultaneous action of both states would nullify each other leading to no discernible effects (e.g., Wearden et al., 2007; Bratzke et al., 2017). Our results could potentially be explained by an effect of faster motion on switch closure latency, or vica versa. Since we have used one range of durations in this study, we cannot separate the additive effects that would be induced by switch closure latency from the proportional effects that would result from clock speed effects. Comprehensively elucidating an additive switch-based effect on perceived time veiled in our data remains a fertile methodological challenge for future research.

The behavioral effects observed in this study can also be readily accounted for by the neural energy model of timing (Pariyadath and Eagleman, 2007, 2008) since based on prior work (e.g., Kaufmann et al., 2000) observing faster walking speeds would be expected to lead to stronger neural activation (i.e., more neural processing) which, in the light of the neural energy model, would lead to longer time estimates. The experimental design and tools utilized in the current study, however, cannot distinguish between these two different theoretical accounts. On the other hand, the lack of an effect of walking direction on perceived time contradicts our hypothesis that was derived from the embodied cognition perspective. According to the embodiment perspective, cognition regarding real-world objects is time-pressured and is body-oriented (Anderson, 2003). We rarely interact with backward walking motion in real life and are therefore less familiar with it compared to forward (i.e., regular) walking. Based on this rationale, we expected the effect of walking speed to be more prominent in the forward walking condition than the backward walking condition, which was not the case, even though participants were indeed able recognize a biologically plausible backward motion compared to an implausible one (see *Experiment 3, Stimulus Rating*). Briefly, the lack of an effect of walking direction in the light of the previous studies does not support the embodied cognition account of our findings.

On a different level, our results can be interpreted from two perspectives; one view assumes that temporal and spatial information processing are independent and the other view assumes that temporal and spatial information processing can be coupled. According to the first approach, one can think that the effect of visual stimulus (such as the flickering presentation of visual input) would be via the stimulus-dependent arousal-based modulation of the central clock mechanism as discussed above (e.g., Droit-Volet and Wearden, 2002). The second approach is supported by work that shows that time perception can be modulated by adaptation to visual properties in a spatially localized fashion, which points at the effects at the level of sensory information processing (e.g., Johnston et al., 2006).

Although our study does not allow us to differentiate between these two accounts, as part of the second theoretical framework, one can speculate regarding the possible neural mechanisms that mediate the modulation of time perception by the observed walking speed (i.e., biological motion). One of the possibilities is that these effects are mediated by the “When Pathway” containing the right parietal cortex (i.e., inferior parietal lobe-IPL) that is assumed to process event timing bilaterally in the visual field (Battelli et al., 2007). Parietal lobe has indeed been shown to be associated with event timing in both monkey (Leon and Shadlen, 2003; Morrone et al., 2005 for LIP involvement) and human work (Husain et al., 1997). In this pathway, visual information is assumed to be relayed from V1 to the middle temporal visual area (MT), that is involved in the perception of speed and direction of motion (Krekelberg and van Wezel, 2013; Liu and Newsome, 2005- irrespective of it being biological or not). Motion-related information from MT is then relayed to multiple areas including the right inferior parietal lobe (IPL), right angular gyrus, supramarginal gyrus, and posterior superior temporal sulcus (Battelli et al., 2007). From the types of motions, the “biological motion” has been argued to be one of the most prominent signals that is processed in this high-level attention-based system that contains the IPL as well as other areas such as the posterior superior temporal sulcus (Grossman et al., 2000, 2005; Grossman and Blake, 2002; Battelli et al., 2003, 2007). It might be this very neural pathway and its functional overlaps through which the observed biological motion and time might interact so robustly as in the case of our work.

Motion-related signals (biological or not) from MT modulate the activity of neuronal populations also in the lateral intraparietal area (LIP – Mazurek et al., 2003), which in and of itself has been shown to be involved in interval timing (e.g., Leon and Shadlen, 2003; Janssen and Shadlen, 2005; Jazayeri and Shadlen, 2015). If timing mechanisms are similar to those in perceptual decision making (e.g., Simen et al., 2011; Balcı and Simen, 2016), where MT neurons are associated with momentary motion whereas LIP neurons integrate those motion-related signals over time (as in the context of two alternative random dot motion discrimination), one would expect the rate of temporal integration to be closely coupled with the speed of observed motion. This constitutes another neural pathway that might mediate the effect of observed motion (biological or not) on perceived time. Note that these arguments have been typically made in relation to the timing of relatively short intervals and their extension to longer scale time such as those utilized in our study requires further work (e.g., see Coull and Nobre, 1998; Bruno and Cicchini, 2016).

Another potential mechanism for the modulation of time perception by biological motion is through the premotor frontal areas that have been implicated both in biological motion (Saygin, 2007) as well as time perception (e.g., Mita et al., 2009). Within this framework, the connections between superior temporal sulcus and premotor areas (e.g., Luppino et al., 2001; Yoshida et al., 2011) might support the modulation of perceived time by the speed of biological motion. Lastly, the temporo-occipital junction (TOJ) has been shown to be activated by unfamiliar compared to familiar walking scenes (Maffei et al., 2014). The lack of an effect of walking direction in the current study suggests that the TOJ is probably not recruited with regard to the interaction between perceived walking speed and perceived time, narrowing down the possible neuroanatomical basis of the effect. Future neuroimaging and neuromodulation studies would help differentiate between these different implementational possibilities.

Weber’s ratio, in its simplest form, has been suggested to be constant when timing different durations with a constant (i.e., non-modulated) clock-speed (Gibbon, 1977; Grondin, 2001), except for very short (Getty, 1975) or very long durations (Bizo et al., 2006); a range of which does not closely bound the durations used in the current study. Simulations conducted based on the decision rules as outlined in Wearden and Ferrara (1995) and the linear modulation of Poisson clock speed by the walking speed showed that WR should remain nearly constant for all walking speeds. Consistent with this prediction, the Weber’s Ratios were relatively constant across conditions in *Experiments 1* and *3*, however, it increased as function of walking speed in *Experiment 2*. Therefore, our findings regarding WRs also supported the predictions of the clock-speed modulation account of the effect of observed walking speed.

In all of our experiments, we modulated *fps* values in order to increase/decrease the speed at which the stick-figure seemed to move. Importantly, a higher *fps* stimulus (our fast walking condition) by definition employs more frames that are presented to the participant per unit time. In relation to theories of timing that emphasize “perceived change per unit time” as the fundamental index of perceived duration (Poynter, 1989), it can be argued that it wasn’t the high speed of movement *per se* that altered perceived durations in our paradigm, but rather the number of frames perceived by the participant per unit time. However, given that all of the simulation videos used in our study were presented with upward of 24 frames per second, beyond which most participants perceive continuous motion (e.g., Condon and Ogston, 1966; Haggard and Isaacs, 1966), such an argument seems implausible. Nonetheless, this possibility could be tested for by keeping the frame rate constant (e.g., 50 *fps*) among speed conditions in a future study.

Our experimental manipulation of walking speed was implemented in a fashion isolated from other visual correlates at the background scene (i.e., the rate of change in the background visual scene). This limited the ecological validity of the stimulus manipulation since natural visual processing of objects typically occurs in the presence of complex backgrounds. An experimental design that contains conditions with (a) a constant walking speed coupled with different rates of change in the background visual scene, (b) the rate of change in the background visual scene congruent with the change in walking speed, and (c) the rate of change in the background visual scene incongruent with the change in walking speed would allow future research to capture the differential effect of rate of change in the visual scene on time perception. In such experimental settings, we would expect the observed effects on time perception to be enhanced in visually congruent conditions and diminished in incongruent conditions provided that the participants process the scene (e.g., visual flow) together with the figure. Under this rationale, for a constant walking speed the rate of change in the background visual scene could also be an independent determinant of alterations in time perception. However, given the object-based visual attentional processing and the fact that various brain regions are differentially involved in the processing of biological and non-biological motion (e.g., Grossman et al., 2000), we would also expect the walking speed of the attended agent to have the dominant modulatory effect on time perception. Lastly, the current study employed no eye-tracking-based visual restrictions throughout the trials so as not to prevent voluntary exploration of the stimuli during the timing of presented intervals. Saccadic eye movements are known to affect perceived time (Yarrow et al., 2001; Morrone et al., 2005; Burr et al., 2010; Suzuki and Yamazaki, 2010; Karşılar and Balcı, 2016; Penney et al., 2016) and therefore future studies could test similar effects to ours by forcing some type of foveal fixation either at the center of the stimulus, or allow for fixations only within the area encompassed by the size of the presented videos. Future studies with such experimental designs are needed to complement our understanding of the effect of observed motion on time perception.

Relatedly, all of our experiments employed stimuli depicting a simple walking motion performed by an animated human-like agent, none of which showed an effect of walking direction on perceived time. As mentioned above, biological plausibility is possibly linked to the mechanism by which an object is timed. Therefore, a future study that tests how self-governing, non-biological motion stimuli (as opposed to backward movement used in the current study) are timed in contrast to stimuli depicting biological motion (i.e., walking), could further elucidate the mechanism by which this modulation of time perception was achieved in the current study. As such, it is possible that the backward walking motion used in our experiments failed to tap into the mechanism by which non-biological/unnaturally moving stimuli are processed (Maffei et al., 2014), which is why it might have exerted no discernible effect on perceived time, as opposed to what was hypothesized. We find such an investigation particularly relevant to our overarching research question since the motor system would be more likely to imitate the biological motion due to higher structural overlap between the human motor system and the observed stimulus (for detailed discussion see Wilson, 2001; Shiffrar and Heinen, 2011) and thereby better extract information regarding the motion-related state of the observed stimulus as a result of its stronger embodiment (Loula et al., 2005). Neuroscientific evidence in related fields further bolsters the relevance of addressing this issue as the brain areas (e.g., premotor areas and cerebellum) that have been implicated in the processing of human movement (Stevens et al., 2000; Saygin et al., 2004; Saygin, 2007) are also known to be involved in interval timing (Merchant et al., 2013).

As a final note, most types of biological motion used in experimental settings, -including many different forms of walking- are typically represented either by video recordings of actual actors, or by point-light animations (Johansson, 1973) which present the action in terms of coherently moving nodes/joints (see Grosbras et al., 2012 for a comprehensive review). While stimuli composed of video recordings benefit from high fidelity in terms of biological plausibility of the observed motion, these types of stimuli suffer from potential embodiment-related confounds depending on the (dis)similarity between the actor and the timing agent. On the other hand, point-light animations (e.g., Saygin et al., 2004; Watanabe, 2008) sidestep this problem by utilizing a more “symbolic” and flexible expression of biological motion with an otherwise invisible actor projected over a static background, which effectively omits all potential confounds such as color, shape, preconceived biases etc. However, point-light stimuli arguably lack some ecological validity, since timing of motion stimuli entails perception of almost all aspects of the observed organism and not just a sub-component of implied coherent motion vectorized in terms of moving dots. The stimuli used in the current study were, in principle, closer to point-light walker animations compared to video recordings; yet unlike their counterpart, they concretely and visibly represented the human motion in its entirety, including the action of the limbs, torso and the head (see Supplementary Materials for animations). To the best of our knowledge, these types of stick-figure stimuli have never been utilized in the context of timing. This methodological novelty, compounded by the relatively prominent effects, which parametrically increase with the experimental manipulation of observed walking speed, put forth the possibility for future studies to employ other similar forms of animation, (including 3-dimensional stimuli embedded within virtual or augmented reality environments), which in turn could more accurately elucidate the mechanism by which observing (or interacting with) some form of biological or non-biological motion could exert its effects on how humans perceive the “flow” of time.

## Data Availability Statement

The datasets analyzed for this study can be found in the Drive Folder URL: https://tinyurl.com/ycc8cgkr.

## Author Contributions

HK, YK, and FB contributed equally to the conception and design of the study. HK and YK prepared the stimuli and collected the data. HK organized the database. HK and FB performed the statistical analyses. All authors contributed to the first and revised draft of the manuscript, read, and approved the submitted version.

## Conflict of Interest Statement

The authors declare that the research was conducted in the absence of any commercial or financial relationships that could be construed as a potential conflict of interest.

## Supplementary Material

DATA SHEET S1 The model comparison tables for all Bayesian analyses conducted.

PRESENTATION S1 The stimuli used in all experiments.
