# Supplementary material for: Dilation and Constriction of Subjective Time Based on Observed Walking Speed
Source: Front Psychol. 2018 Dec 21;9:2565. doi: 10.3389/fpsyg.2018.02565 (PMC6309241; doi:10.3389/fpsyg.2018.02565)
Supplement: DATA SHEET S1 — The model comparison tables for all Bayesian analyses conducted. [file Data_Sheet_1.PDF]

Supplementary Material

Dilation and Constriction of Subjective Time Based on Observed Walking Speed

Hakan Karşılar[1], Yağmur Deniz Kısa[1] & Fuat Balcı[1,2]*

[1]Koç University, Department of Psychology, Istanbul, Turkey

[2]Koç University Center for Translational Medicine, Istanbul, Turkey

*** Correspondence:**
Fuat Balcı, Ph.D.
fbalci@ku.edu.tr

1 Supplementary Bayesian Analyses Tables

The detailed output tables from the Bayesian analyses conducted in the original study are presented below. Results of identical analyses performed with walking *direction* and walking *speed* as the independent variables, and *Point of Subjective Equality* or the *Weber's Ratio* as the independent variable are reported in separate sections below.

1.1 Experiment 1

Dependent Variable: Point of Subjective Equality

Model Comparison

**Models**	**P(M)**	**P(M|data)**	**BF $_M$**	**BF $_{10}$**	**error %**
Null model (incl. subject)	0.200	1.682e -14	6.726e -14	1.000	
Speed	0.200	0.751	12.067	4.466e +13	0.713
Direction	0.200	2.718e -15	1.087e -14	0.162	1.016
Speed + Direction	0.200	0.129	0.593	7.681e +12	1.967
Speed + Direction + Speed ✻ Direction	0.200	0.120	0.544	7.124e +12	1.558

Note. All models include subject.

Analysis of Effects

**Effects**	**P(incl)**	**P(incl|data)**	**BF $_{Inclusion}$**
Speed	0.600	1.000	3.412e +13
Direction	0.600	0.249	0.221
Speed ✻ Direction	0.200	0.120	0.544

Dependent Variable: Weber's Ratio

Model Comparison

Models	P(M)	P(M|data)	BF M	BF 10	error %
Null model (incl. subject)	0.200	0.487	3.801	1.000	
Speed	0.200	0.093	0.409	0.190	1.283
Direction	0.200	0.339	2.054	0.696	3.082
Speed + Direction	0.200	0.066	0.284	0.136	2.375
Speed + Direction + Speed ✻ Direction	0.200	0.014	0.059	0.030	1.971

Note. All models include subject.

Analysis of Effects

Effects	P(incl)	P(incl|data)	BF Inclusion
Speed	0.600	0.174	0.140
Direction	0.600	0.420	0.483
Speed ✻ Direction	0.200	0.014	0.059

1.2 Experiment 2

Dependent Variable: Point of Subjective Equality

Model Comparison

Models	P(M)	P(M|data)	BF M	BF 10	error %
Null model (incl. subject)	0.200	5.700e -39	2.280e -38	1.000	
Speed	0.200	0.708	9.681	1.241e +38	1.190
Direction	0.200	1.160e -39	4.640e -39	0.204	3.075
Speed + Direction	0.200	0.263	1.427	4.613e +37	5.029
Speed + Direction + Speed ✻ Direction	0.200	0.029	0.121	5.161e +36	2.975

Note. All models include subject.

Analysis of Effects

Effects	P(incl)	P(incl|data)	BF Inclusion
Speed	0.600	1.000	∞
Direction	0.600	0.292	0.275
Speed ✻ Direction	0.200	0.029	0.121

Dependent Variable: Weber's Ratio

Model Comparison

Models	P(M)	P(M|data)	BF M	BF 10	error %
Null model (incl. subject)	0.200	0.143	0.666	1.000	
Speed	0.200	0.694	9.084	4.865	1.174
Direction	0.200	0.023	0.094	0.160	2.265
Speed + Direction	0.200	0.107	0.478	0.748	1.572
Speed + Direction + Speed ✻ Direction	0.200	0.033	0.138	0.234	2.247

Note. All models include subject.

Analysis of Effects

Effects	P(incl)	P(incl|data)	BF Inclusion
Speed	0.600	0.834	3.360
Direction	0.600	0.163	0.130
Speed ✻ Direction	0.200	0.033	0.138

1.3 Experiment 1 and 2 Comparison of PSE values

Model Comparison

Models	P(M)	P(M|data)	BF_M	BF_{10}	error %
Null model (incl. subject)	0.053	2.357e -62	4.243e -61	1.000	
Speed	0.053	1.230e -14	2.213e -13	5.216e +47	1.443
Direction	0.053	2.909e -63	5.237e -62	0.123	1.168
Speed + Direction	0.053	1.669e -15	3.004e -14	7.080e +46	1.381
Speed + Direction + Speed ✻ Direction	0.053	3.126e -16	5.627e -15	1.326e +46	1.452
Test_Subject	0.053	5.204e -63	9.368e -62	0.221	1.534
Speed + Test_Subject	0.053	3.542e -15	6.375e -14	1.502e +47	0.985
Direction + Test_Subject	0.053	6.359e -64	1.145e -62	0.027	2.040
Speed + Direction + Test_Subject	0.053	4.960e -16	8.927e -15	2.104e +46	1.439
Speed + Direction + Speed ✻ Direction + Test_Subject	0.053	9.481e -17	1.707e -15	4.022e +45	2.168
Speed + Test_Subject + Speed ✻ Test_Subject	0.053	0.792	68.590	3.360e +61	1.462
Speed + Direction + Test_Subject + Speed ✻ Test_Subject	0.053	0.118	2.410	5.009e +60	2.382
Speed + Direction + Speed ✻ Direction + Test_Subject + Speed ✻ Test_Subject	0.053	0.033	0.606	1.382e +60	2.704
Direction + Test_Subject + Direction ✻ Test_Subject	0.053	1.329e -64	2.393e -63	0.006	1.693
Speed + Direction + Test_Subject + Direction ✻ Test_Subject	0.053	1.524e -16	2.743e -15	6.464e +45	3.896
Speed + Direction + Speed ✻ Direction + Test_Subject + Direction ✻ Test_Subject	0.053	2.984e -17	5.370e -16	1.266e +45	2.772
Speed + Direction + Test_Subject + Speed ✻ Test_Subject + Direction ✻ Test_Subject	0.053	0.043	0.818	1.844e +60	3.306
Speed + Direction + Speed ✻ Direction + Test_Subject + Speed ✻ Test_Subject + Direction ✻ Test_Subject	0.053	0.012	0.221	5.139e +59	6.762
Speed + Direction + Speed ✻ Direction + Test_Subject + Speed ✻ Test_Subject + Direction ✻ Test_Subject + Speed ✻ Direction ✻ Test_Subject	0.053	0.002	0.029	6.903e +58	13.139

Note. All models include subject.

Analysis of Effects

Effects	P(incl)	P(incl|data)	$BF_{Inclusion}$
Speed	0.737	1.000	∞
Direction	0.737	0.208	0.094
Test_Subject	0.737	1.000	2.494e +13
Speed ✻ Direction	0.316	0.046	0.105
Speed ✻ Test_Subject	0.316	1.000	1.169e +14
Direction ✻ Test_Subject	0.316	0.057	0.131
Speed ✻ Direction ✻ Test_Subject	0.053	0.002	0.029

1.4 Experiment 3

Dependent Variable: Point of Subjective Equality

Model Comparison

Models	P(M)	P(M|data)	BF_M	BF_{10}	error %
Null model (incl. subject)	0.200	4.109e -31	1.644e -30	1.000	
Speed	0.200	0.778	14.015	1.893e +30	0.896
Direction	0.200	7.732e -32	3.093e -31	0.188	2.082
Speed + Direction	0.200	0.186	0.915	4.532e +29	2.777

Model Comparison

Models	P(M)	P(M|data)	BF $_M$	BF $_{10}$	error %
Speed + Direction + Speed ✻ Direction	0.200	0.036	0.149	8.714e +28	2.040

Note. All models include subject.

Analysis of Effects

Effects	P(incl)	P(incl|data)	BF $_{Inclusion}$
Speed	0.600	1.000	6.005e +15
Direction	0.600	0.222	0.190
Speed ✻ Direction	0.200	0.036	0.149

Dependent Variable: Weber's Ratio

Model Comparison

Models	P(M)	P(M|data)	BF $_M$	BF $_{10}$	error %
Null model (incl. subject)	0.200	0.462	3.436	1.000	
Speed	0.200	0.259	1.400	0.561	0.879
Direction	0.200	0.143	0.667	0.309	1.084
Speed + Direction	0.200	0.085	0.370	0.183	4.534
Speed + Direction + Speed ✻ Direction	0.200	0.051	0.215	0.111	2.734

Note. All models include subject.

Analysis of Effects

Effects	P(incl)	P(incl|data)	BF $_{Inclusion}$
Speed	0.600	0.395	0.435
Direction	0.600	0.279	0.258
Speed ✻ Direction	0.200	0.051	0.215
